# Fungal metabarcoding data integration framework for the MycoDiversity DataBase (MDDB)

**DOI:** 10.1515/jib-2019-0046

**Published:** 2020-05-28

**Authors:** Irene Martorelli, Leon S. Helwerda, Jesse Kerkvliet, Sofia I. F. Gomes, Jorinde Nuytinck, Chivany R. A. van der Werff, Guus J. Ramackers, Alexander P. Gultyaev, Vincent S. F. T. Merckx, Fons J. Verbeek

**Affiliations:** Leiden Institute of Advanced Computer Science (LIACS), Leiden University, Leiden, The Netherlands; Understanding Evolution, Naturalis Biodiversity Center, Leiden, The Netherlands; Department of Evolutionary and Population Biology, Institute for Biodiversity and Ecosystem Dynamics, University of Amsterdam, Amsterdam, The Netherlands

**Keywords:** biogeography, data integration, DNA barcoding, environmental samples, fungal biodiversity

## Abstract

Fungi have crucial roles in ecosystems, and are important associates for many organisms. They are adapted to a wide variety of habitats, however their global distribution and diversity remains poorly documented. The exponential growth of DNA barcode information retrieved from the environment is assisting considerably the traditional ways for unraveling fungal diversity and detection. The raw DNA data in association to environmental descriptors of metabarcoding studies are made available in public sequence read archives. While this is potentially a valuable source of information for the investigation of Fungi across diverse environmental conditions, the annotation used to describe environment is heterogenous. Moreover, a uniform processing pipeline still needs to be applied to the available raw DNA data. Hence, a comprehensive framework to analyses these data in a large context is still lacking. We introduce the MycoDiversity DataBase, a database which includes public fungal metabarcoding data of environmental samples for the study of biodiversity patterns of Fungi. The framework we propose will contribute to our understanding of fungal biodiversity and aims to become a valuable source for large-scale analyses of patterns in space and time, in addition to assisting evolutionary and ecological research on Fungi.

## 1 Introduction

The kingdom Fungi is a major group of organisms. Fungi play essential roles in ecosystems and their ecological importance is widely studied. They are involved in the decomposition of organic carbon and contribute to the transformation of phosphorus and nitrogen compounds into crucial resources for the life of other organisms [1−3]. Fungi are found living under an extremely wide variety of environmental conditions and they have evolved diverse feeding strategies; symbiotic (i. e., intimate interactions between living partners, such as bacteria [4], plants [5], [6] and animals [7], [8]), parasitic [9] and saprotrophic (whereby fungi decompose dead organic material) [10].

There are currently between 120,000 and 144,000 species [11], [12] of fungi described, but it has been estimated that the actual diversity of the group includes millions of species [13]. Yet, due to the complexity of their morphological structures, their dependencies on other organisms, and their cryptic lifestyles, much of their global distribution and diversity remains to be documented. Furthermore, the environmental factors controlling the spatial patterns of fungi are still poorly understood. Only with the advent of DNA-based molecular techniques, have researchers been able to look closely at the ecological forces that structure fungal communities. In particular, nowadays researchers make use of metabarcoding, a technique which combines high-throughput DNA sequencing methods (HTS) with a common barcode gene to identify all species present in environmental samples.

This approach has revealed new fungal species at a high rate [14], [15] and provides critical new insights to assessments on fungal diversity and the distribution patterns of fungal communities. For example, numerous metabarcoding studies on fungal diversity have been based entirely on DNA extracted from soil, a habitat of high fungal diversity [16], [17]. Raw DNA sequencing data from individual studies, in addition to descriptive information of the collected samples, host environments, and locations are now stored in accessible sequence read archives, for example in the National Center for Biotechnology Information (NCBI) Sequence Read Archive (SRA) [18]. These archives are therefore potential powerful sources of information for extending our knowledge of fungal distributions across multiple geographic and ecological scales. However, the metadata associated to samples is heterogeneous and information of interest is not directly accessible from the raw DNA data, hence data from individual studies are not directly comparable. These obstacles prevent large scale assessments for fungal biodiversity and distribution based on DNA data retrieved from public repositories. Therefore, here we introduce the MycoDiversity DataBase (MDDB), a curated repository of public integrated environmental samples for assisting the studies on fungal biodiversity.

In the MDDB, processed data from SRA is stored and integrated such that fungal DNA sequences are directly retrievable from a study source. MDDB facilitates navigation from individual environmental sample to a larger scale by linking the spatial components of the origins of the samples. Even though Fungi are ubiquitous in all ecosystems [19], their abundance and community structure vary across biomes and are influenced by the abiotic and biotic characteristics of their habitats. With the MDDB, a set of fungal sequences observed in refined environmental criterias can be obtained, for example fungal communities detected in very acidic soils, and researchers can explore how communities differ along particular habitat gradients. The MDDB incorporates information retrieved from public resources, including literature, SRAs and taxonomic sources used for identifying sequencing data. The integration of these data is implemented by applying three methodologies. The first one is the extensive curation applied to the annotations retrieved from the data repositories. The second is the generation of mappings for the association of data retrieved from the different data repositories. The last is the application of a uniform processing method adapted to the raw DNA data retrieved from the sequence archives. This latter method allows the incorporation of environmental traits obtained from samples and fungal DNA sequences, and thus enables to integrate data from different studies. With the inclusion of metabarcoding studies, MDDB allows to investigate the relationships of Fungi within their ecosystems, in addition to explore which are the environmental factors controlling their spatial patterns.

Our database has the objective of becoming a supportive tool for researchers to gain a better insight of the actual biodiversity of these organisms, and in addition, to facilitate the identification of the ecological fundamental features driving fungal distribution patterns over large geographic scales.

## 2 Background information

### 2.1 Fungal diversity

Traditionally, mycologists have been describing and classifying organisms belonging to this eukaryotic group by using morphological traits, mostly of sporocarps, the macroscopic reproductive structures present in some lineages of Fungi. This classical approach has severe limitations. For instance, many species belonging to arbuscular mycorrhizal fungi do not form visible fruiting structures [20], seasons also have an impact on sporocarp production [21] and the identification of certain groups may be restricted during their non-fruiting stages [22]. However, the recent adoption and dissemination of DNA-based molecular tools has greatly reduced the barriers to sampling and identifying fungi from fungal hyphae. The sequencing methods not only refined taxonomic relationships hypotheses which were based on morphological evidence, but also assisted the rapid identification of novel taxa [23].

The DNA barcoding technique is now a widespread sequencing approach used to identify species [23], [24], raising the actual diversity of the group to a predicted 5.1 million of species of fungi [25]. The highly variable nuclear ribosomal internal transcribed spacer (ITS) region is proposed as the primary fungal DNA ‘barcode’ marker for species identification [26]. HTS technologies has transformed our perspective on fungal distribution by enabling the detection of organisms directly from their host environments. Barcode sequences detected in environmental samples can be simultaneously and rapidly sequenced by using a variety of HTS platforms [27], [28], [29].

### 2.2 HTS technologies on fungal diversity

In this past decade, there has been an increasing number of environmental studies on fungal communities which relied on data obtained from HTS platforms. Fungal diversity in ecosystems have been studied over different geographical scales, such as on small-scale [30−35] and on global-scale [36], [37], [38]. Other studies focused on environmental aspects [39], [40], [41] or on the investigations of fungal-host relationships [42], [43], [44], [45] and for observing effects of climatological gradients on biodiversity [46].

Such investigations should be carried out by applying the following criterias: the same sampling protocol for the collected samples, the equivalent HTS platform used for sequencing DNA barcode data and a uniform method for processing the raw HTS data.

### 2.3 Repositories for HTS data

The SRAs of the International Nucleotide Sequence Database Collaboration (INSDC) [47] are the European Bioinformatics Institute (EMBL-EBI) European Nucleotide Archive (ENA) [48], the NCBI SRA and the DNA DataBank of Japan Sequence Read Archive (DRA) [49]. These archives have been developed for providing the huge amount of data generated from HTS technologies in form of files containing sequence reads. As of end of April 2019, SRA contained more than 10 petabasepairs (10ˆ16 basepairs) of open-access HTS data [50] linked to almost 200,000 published studies, in which over 4000 SRA records belong to environmental metabarcoding studies.

### 2.4 Processing HTS material for fungal detection

The raw sequencing data generated from HTS has its computational challenges for obtaining the fungal composition within an environmental sample. In order to provide species-level data for community analysis and ecological studies, the amplicon reads generated by HTS technologies need to be categorized in distinct units, which are a proxy for fungal species. Numerous bioinformatics algorithms [51], [52], [53], [54], [55], [56], [57] have been developed to cluster the raw reads based on their sequence similarity into representatives of roughly species-level, commonly referred to Operational Taxonomic Units (OTUs). The amplicon reads contain errors which are generated during the sequencing [27], [58], [59], [60], [61]. A quality filtering step [62], [63], [64] can be applied to remove the errors from amplicon reads before the clustering procedure into OTUs.

### 2.5 Sequence based taxonomic identification

The taxonomic annotation of OTUs relies on sequence similarity searches in reference databases. For sequence similarity searches, BLAST [65] is the most common tool. In the context of the fungal kingdom, UNITE [66] is considered as the main reference ITS database for the identification of fungi [67]. UNITE groups the ITS sequences from specimen/culture vouchers collected at several sequence similarity thresholds to obtain species-level OTUs referred as species hypotheses. All SHs (458,797 as of August 2018) [68] have a unique digital object identifier (DOI) which provides a standardized documentation of which taxa were found, and promotes unambiguous reference communication across studies [15].

### 2.6 Integration of published sequence data

Many studies rely on the use of HTS techniques for fungal assessment, but due to the different HTS platforms and the heterogeneity of the HTS data generated, the curation of these data is laborious and an uniform strategy needs to be applied. Consequently, there are very few integrated studies based on public repository data. In addition, due to the enormous species diversity of this group of organisms, many studies only focus on particular fungal groups for large-scale assessments [69].

Large-scale biogeographic signals based on HTS data are still scarce. This is mostly due to the lack of standardized approaches for extrapolating the HTS raw data. Even if guidelines do exist [14], [70], different researches apply yet different methods for denoising HTS data and decisions on OTU assignments. Not only this may affect the diversity estimate, but the direct comparison of OTUs among studies is restricted. Meiser et al., [71] were the first to exhibit a comparative metabarcoding analyses of public metabarcoding studies. Even though their approach considered only three studies deposited in SRA, the uniform denoising method applied on HTS public data, retrieved from SRA, allowed the integration of studies such to compare fungal communities on the basis of ITS sequences.

### 2.7 Databases on fungal sequence data

There have been previous attempts in building DNA-based databases taking into account fungal DNA data and associate it to information on their environment. For example, in 2010, the MaarjAM database was released [72], a repository of reference sequences belonging to the mycorrhizal fungi Glomeromycota. The database associates information about geography, habitat and climate to Glomeromycota barcode sequences. These barcodes are clustered in ‘Virtual Taxa’, a proxy for fungal species. However, the data deposited in MaarjAM does not include SRA data and it is only limited to published processed sequence data assigned to Glomeromycota obtained in GENBANK [73]. Several studies describing Glomeromycota have included their data in SRA (e. g., SRA study accession numbers: SRP066844, SRP087758, SRP075244, SRP067281, SRP070752). These studies reveal relationships between environmental gradients and Glomeromycota richness covering many ecosystems. MaarjAM’s environmental metadata is maintained by a group of specialists who extrapolate the information from literature so that provide high quality annotation. The incorporation of new publications and sequences in the database has decreased in the past years (two publications in the years 2018–2019). This is due to the non-automated approach of curating records and the clustering method applied which has been criticized [74].

## 3 Materials and methods

### 3.1 Data acquisition

**Figure 1:**
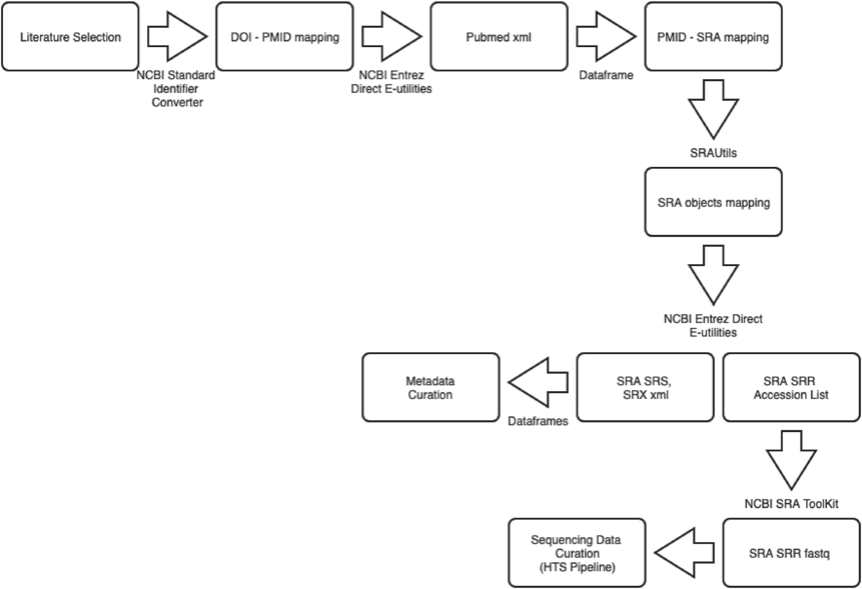
Data acquisition workflow for retrieving data from Pubmed and SRA repositories. Once the data is retrieved, the final step of the data acquisition involves the curation of the two data types, the metadata retrieved from PubMed and SRA (Metadata Curation), and of the raw HTS data of the SRA fastq files (Sequencing Data Curation).

The MDDB aims to provide a quality-controlled repository of public metabarcoding studies related to fungal diversity. The publications containing the raw HTS data submitted to sequence archives, were used for inclusion in MDDB. The workflow of the data acquisition methodology is summarized in Figure 1. Rather than selecting data directly from public studies, our approach was to investigate whether data could be retrievable from publications which describe studies. For the development of MDDB, our starting point was the selection of 25 publications (Appendix A) related to fungal metabarcoding studies thus by using the keywords ‘ITS’, ‘fungi’ and ‘metabarcoding’. This selection all included DOIs and were all associated to HTS data.

The subsequent step involved the PubMed [75] mappings to the DOI list. We have used the NCBI standard identifier converter API [76] for retrieving the list of Pubmed identifiers of the publications. The list of Pubmed identifiers was used to obtain the PubMed records in a XML format by means of a Biopython wrapper (v.1.73) [77] on the NCBI Direct Entrez E-utilities package [78]. The records in PubMed contain formalized annotations including the abstract, authors and affiliations, and crossed-reference identifiers to other repositories. An XML parser in Python (v.2.7.15) was used to retrieve the elements and store them in a Python structure DataFrame. As from 2014, the sequence read archives of INSDC and the NCBI BioSample and BioProject databases [79] are included in the DataBankList PubMed element [80]. This element corresponds to a data source for which the accession number list of sequence data can be provided. The name of the reference repositories and their values are retrieved automatically and give rise to the PMID – SRAs mapping. For the PubMed records that do not contain this element, the complete Publication-Study mapping is achievable by retrieving the sequence read accession numbers from the publication. Our method is based on searching for regular expressions in PDF files of publications. To that end, we have defined the set of relevant regular expressions. We use PyPDF2 [81] (v.1.26.0), a PDF Python parser to find the matches to such regular expressions. The possible matches are contained in a list of sequence archive prefixes which belong to SRA, ENA and BioProject. When a match is detected, the full string is retrieved. Table 1 illustrates the list of regular expressions to search in the text of the PDF of the publications. Using this strategy, from 25 publications (88%), we detected 22 accession numbers (Appendix A, Article Attribute). From this selection, two articles contain GENBANK identifiers which provide processed HTS data. These were not considered as they were not part of the set of regular expressions of interest and are beyond the scope of the database. Our method could not detect sequence read accession identifiers which were located in tables of the PDF files. This was the case of [71], for which a manual retrieval was applied. For the publications which reference BioProject accession numbers, the Entrez E-utilities allowed to retrieve the associated SRA study accession numbers (SRP).

**Table 1:** Regular expressions used for the detection of the sequence archive prefixes in the text of publications. For each regular expression, we provide the total number of publications for which a match is detected.

Regular Expression	INSDC DB	Number of Publications
SRA	SRA	5
SRP	SRA	8
SRS	SRA	2
SRX	SRA	1
PRJNA	BioProject	5
PRJEB	BioProject	1
ERP	ENA	0
ERS	ENA	0

The majority of the articles we have selected contained SRA as the source of sequence data (Appendix A, Study Attribute), thus this became our primary repository mapping for the related literature and SRA.

The SRA submission object domain, known simply as SRA contains 4 name space mappings, the study of research (SRP), the experiment used for sequencing (SRX), the sample of origin (SRS) and the files generated by the HTS technologies, the sequence run (SRR). In order to automatically retrieve all the object identifiers linked to an SRA object, we have used the SRAUtils (https://bootstrappers.umassmed.edu/guides/main/), a bootstrapper for SRA Run Info CGI [82]. The metadata linked to SRP, SRX and SRS objects have been retrieved with E-search and E-fetch of the NCBI Direct E-utilities. As for the SRR objects, the raw sequence data (provided in standard flow gram format files) generated by HTS platforms, are retrieved and converted in Sanger FASTQ format by using the prefetch and fastq-dump (v.2.9.0) of the NCBI SRA Toolkit [83]. By providing in input the DOI of a publication, the data acquisition pipeline (https://github.com/naturalis/mycodiversity/tree/master/ncbi_data_acquisition) retrieves metadata associated to the SRA submission of a metabarcoding study including data linked to the NCBI BioProject and BioSample databases. The pipeline was conducted over the DOIs of the publications listed in Appendix A by using an Intel(R) Core(TM) i7-4770HQ Processor, CPU @ 2.20 GHz, 16 GB mac OS v.10.13.6 17G65 machine. As an indication, running the pipeline on one single publication which contains the PMID cross reference to SRA [44], takes 00:03:35 min for retrieving data. The data retrieved includes metadata belonging to the SRA submission mappings, 166 BioSamples, 166 experiments, mappings to the sequence run files (SRRs) and to Literature. The five files (393 KB) are all saved in a CSV format. We have compiled all the libraries in the GitHub repository (https://github.com/naturalis/mycodiversity/blob/master/ncbi_data_acquisition/requirements.txt).

### 3.2 Data curation

#### 3.2.1 Metadata curation and enrichment

The annotations retrieved from the resources were stored in Python DataFrames for which we have assigned categories. The ‘Literature’ category stores annotations acquired from the PubMed source, ‘Study’ contained description of the experiment conducted, the sample source, and the purpose of the research study. In ‘Sequence’, we have stored the run read files fetched from SRR objects.

When metadata associated to studies use standardized vocabularies, the study integration improves and the interpretation across studies is possible. Significant guidelines [84], [85], [86], are providing extensive emphasize on the application of formats, metadata standards and ontology based vocabularies for leading to knowledge integration and data reusability. Researchers who share data are aware of the importance of following standard procedures [85], [86], [87], [88], [89] but assigning controlled term vocabularies to the data submitted is still lacking [90]. Though SRA to be submitted requires recommended formats and standards for metadata fields, we have seen inconsistency in the format and quality in the selected studies. Because of this data heterogeneity, we have extended the annotation by the use of standard formats and terms for allowing the increase of study comparisons. The date values contained in ‘Literature’ and ‘Study’ categories have been converted to a standard format following the International Organization for Standardization protocol. The motivation of Bernstein et al., [91] to develop MetaSRA, relied on the lack of opportunity to perform large-scale analyses due to the diversity of samples in SRA and the poor structure of the metadata associated with each sample. Though their work has focused on human samples and mapping annotations to terms in biomedical ontologies, their resource shows the great impact on the use of controlled vocabularies and ontologies for the investigations across diverse conditions present in SRA metadata. For our research, it is important to curate the information related to the collected site in order to perform large-scale analyses. We have observed that although controlled vocabularies are supported for the annotation of geographic location site of a sample, and it is highly recommended to provide names from the INSDC dictionary [79], yet the free text insertion is still permitted. We have used Geocoder [92] (v.1.38.1.), a geocoding Python library used for mapping the country terms to standard GeoNames [93] identifiers. The GeoNames API (http://www.geonames.org/export/web-services.html) allowed us to retrieve parent names of the list of GeoNames countries, and these were used to assign a new term ʻcontinentʼ that was linked to the country name. Knowledge of the exact position on the globe where a sample is been collected is a powerful approach to fungal distribution investigation. Many studies deposited in SRA archives provide the geographical coordinates of the sample site collected; however these are in different formats (e. g., degrees or decimal). For allowing the integration of studies by using the coordinate location field in SRA, we have converted all coordinate values to decimal format and split this field into two distinct attributes: latitude and longitude. For including samples that do not provide coordinate values, we have used their GeoNames country identifiers for retrieving the country centroids values and assigned them to the country terms. The Google Maps javascript API (https://developers.google.com/maps/documentation/javascript/reference/) assisted in the correction of coordinate values that did not correspond to the exact spatial location displayed on a world map. This method checks and replaces the incorrect coordinate values with the GeoNames coordinate values provided in GeoNames.

Descriptions of the habitat where the sample has been collected are important for inferring their contributions on a spatial scale diversity. The environmental context, known as biome is one of the most important feature for describing the ecosystem of a sample. It is recommended to assign Environment Ontology (ENVO) [94] terms to the attributes describing the environment. We have used the EXTRACT 2.0 tool [95] for the recognition of the term provided in the SRS metadata and for the retrieval of the corresponding ENVO identifiers. All original sample metadata values are kept as linked reference to data provenance and the values which are curated are used as extended annotation.

#### 3.2.2 HTS data curation and annotation

Because of the heterogeneity and diversity of available pipelines, we have developed a stand-alone pipeline for the generation of OTUs which encounters the diversity of the amplicon data generated by the different HTS platforms. The Processing for Fungal ITS Sequences pipeline (PROFUNGIS) (https://github.com/naturalis/mycodiversity/tree/master/PROFUNGIS) is a pipeline developed for downloading SRA reads (SRR) and for the generation of Zero-radius OTUs (ZOTUs). For historical and technical reasons, OTUs are typically constructed using a clustering threshold similarity of 97% [96], while for ZOTUs, a 100% identity threshold is applied [97]. According to [98], ZOTUs have the advantage of being directly comparable between datasets without the use of reclustering. The application of ZOTUs have shown improvements in reusability and reproducibility, and according to [99], [100], they should replace the 97% OTUs as the standard unit of marker-gene analysis. The pipeline includes the UNOISE (v.3) algorithm [64], a standard algorithm in the mycological and ecological community for performing error correction on amplicon reads. The PROFUNGIS pipeline is executable by providing a set of input parameters. The mandatory arguments include: 1) forward and reverse primers used, 2) the type of ITS subunit marker sequenced 3) the platform and 4) a single SRR accession or set of SRR accessions of SRA. Following the filtering recommended steps of [101], optional parameters are also included: the minimal length overlap (minOverlap) for the merging of the Illumina read sets, the estimated error (maxEE), and the minimal filtering length for trimming. The HTS platforms which PROFUNGIS accepts are Illumina (MiSeq and HiSeq 2000 instruments), 454 (GS Junior, GS FLX Titanium and GS FLX + instruments) and Ion Torrent (PGM). In the case of Illumina platform, PROFUNGIS will include the merging step for creating consensus sequences from the paired-end reads. For the creation of the ZOTUs set, we have included the 454 GS FLX Titanium platform sequence run files belonging to the SRA Study SRP043706. This included >3 million raw reads (3.11 Gb). For the mandatory parameter values, we have provided the following: 1) forward and reverse primers retrieved from the design experimental metadata (SRX642180–SRX642691), 2) ITS2 marker as stated in [36], 3) platform 454 GS, retrieved from (SRX642180–SRX642691) and 4) accession list containing the sequence run accession numbers (SRR1502225–SRR1502736). The default values were kept for the optional parameters, maxEE = 1.0, minOverlap = 60 bp, and minimal filtering length = 250 for both ITS1 and ITS2 subregions of ITS. These values are recommended and are based on experimental tests during the development of PROFUNGIS and on previous studies [101], [64]. UNITE FASTA (v.7.2) release (http://doi.org/10.15156/BIO/587475) was used as a contamination filter and for the taxonomic assignment of the ZOTUs generated from each SRR file. The contamination filter uses BLAST to query the ITS ZOTUs against the UNITE database. This approach allows to remove putative ZOTUs that are not similar to known reference ITS sequences as well as to filter ZOTUs which are not associated to fungi, by using a threshold value of 70% similarity. The PROFUNGIS pipeline can run individually, but also sequentially to the data acquisition pipeline. As an indication, for a subset of 100 SRR accession numbers belonging to the SRP043706 study [36], to be converted into 629,304 (645,8 MB) FASTQ format sequences and processed to 47,529 (12,5 MB) ZOTU FASTA format sequences, PROFUNGIS takes 00:37:33 min. PROFUNGIS runs were performed by using an Intel(R) Core(TM) i7-4770HQ Processor, CPU @ 2.20 GHz, 16 GB mac OS v.10.13.6; and the pipeline can be used on other OS (https://github.com/naturalis/mycodiversity/blob/master/PROFUNGIS/Dockerfile). We have compiled the libraries in the repository (https://github.com/naturalis/mycodiversity/blob/master/PROFUNGIS/requirements.txt).

## 4 Implementation

### 4.1 Database design and implementation

**Figure 2:**
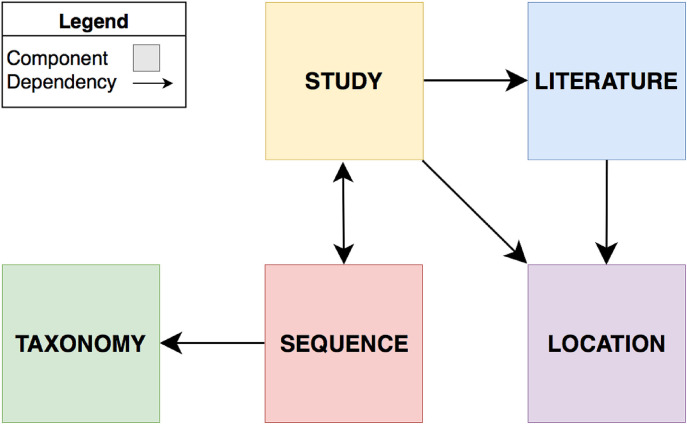
Components and Dependencies of the fungal metabarcoding data integration.

The MDDB is designed for the integration of metadata belonging to metabarcoding studies to processed sequence data obtained from the linked HTS data. Curated extended annotations have been linked to the original annotations for enabling the interoperability among metabarcoding studies which have been described in literature. The logical design of the MDDB has followed the recommended procedure of a conceptual scheme from the entity-relationship (ER) model [102]. The data has been organized and is distributed into the main conceptual components: Study, Sequence, Taxonomy, Literature and Location (Figure 2).

The concepts and information belonging to the components Literature and Study derived from data obtained from NCBI including PubMed, SRA and the BioProject and BioSample databases. Data belonging to the component Sequence is affiliated to information obtained from processed HTS data. The Taxonomy and Location components are reference repositories which further enrich the conceptual scheme by labeling the sequencing data of the Sequence component and assisting the Study and Literature components in the context of geography.

**Figure 3:**
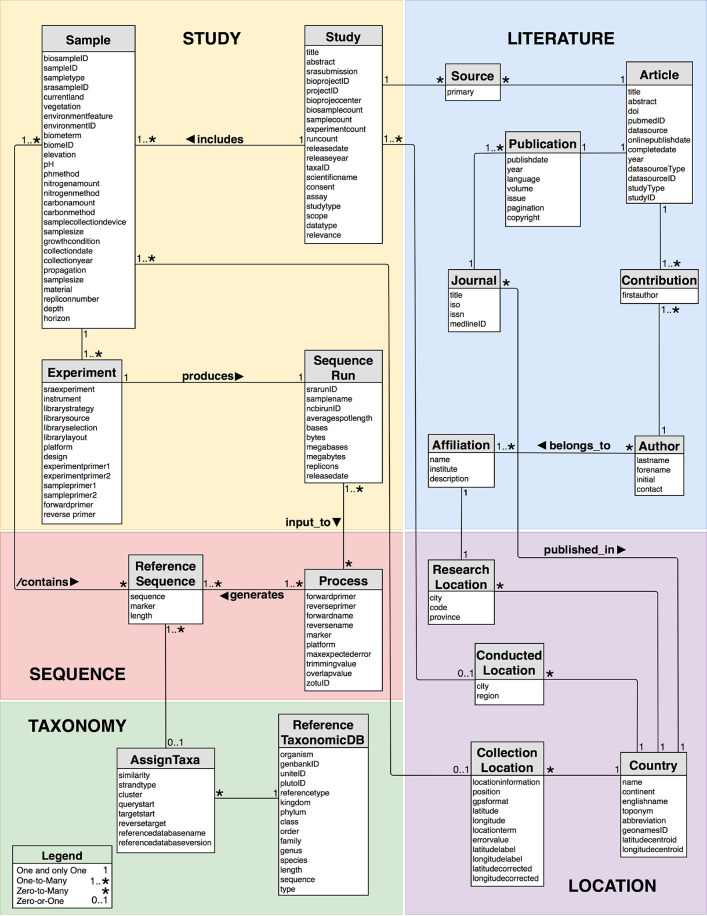
UML data model of the MDDB.

The table structure is depicted in the UML diagram of Figure 3. For the entity tables, unique internal keys, i. e., primary keys, are auto-generated. The provenance mappings of aforementioned literature and of SRA have enabled a creation of relationships within the database. The values contained in the entities Article, Journal, Author and Affiliation of the component Literature, originated from the elements automatically retrieved from PubMed. The ‘Source’ relation contains the mapping of studies as referred in literature and it connects the components Literature to Study. Study contains data retrieved from SRA and it concerns the relationships amongst the SRA objects.

Each sample collected for research purposes is provided in SRA with an SRS accession and it belongs to an individual study, while a study may include several samples (cf. Study.includes in Figure 3). We have observed that SRA studies may not provide sample objects, but HTS data needs to be obtained by a material (i. e., soil). Therefore, as an MDDB constraint, Sample is always defined. The relation ‘Experiment’ thus provides the experimental procedure conducted to a given sample and is the source for connecting the sample composition (i. e., soil) to raw data sequenced produced by an experimental strategy. We store the provenance of the run sequence files because different approaches for processing raw sequences influence the diversity estimates [71]. The PROFUNGIS pipeline has been developed for extracting biological significant sequence information from the SRA SRR files in a uniform manner. The ‘Process’ relation (cf. Sequence.Process in Figure 3) then stores the pipeline parameter values used for the generation of the ZOTUs. The ‘ReferenceSequence’ entity of the Sequence component provides the unique ZOTU representatives incorporated from the processed SRR files generated in experiments. This entity also includes annotation of the barcode markers, i. e., ITS1 and ITS2 subunits, and a sequence length. Post-processing methods, such as clustering and phylogenetic reconstructions, can be applied to this set. The ‘contains’ mapping (cf. Sequence.contains in Figure 3) is a derived relationship and it guarantees the inclusion of the ZOTU reference sequences to the original sample collected and to the data provenance. This relation integrates ZOTUs into the collections of samples provided in MDDB and it is a prompt source for observing the number of shared ZOTUs among environmental conditions and to compare ZOTUs between samples. In addition, the set of ZOTU sequences of ‘ReferenceSequence’ have been blasted against the UNITE reference database (cf. Taxonomy.AssignTaxa in Figure 3) and the mapping provides the blast taxonomic name hit as well as the blast similarity value hit. For the Location component, the location information observed in the Study and Literature components have been related to the geographical territory names and positions by mapping to the referenced country from GeoNames information.

### 4.2 Database deployment

#### 4.2.1 MonetDB

The MonetDB database-management system (DBMS) [103], [104] is an open source column-oriented client-server DBMS which has been particularly designed as a scalable system to be able to deal with large data volumes [105]. To that end MonetDB shows to be extremely useful for data mining applications in astronomy [106], online analytical processing applications [107], geographic information systems [108], [109] and sequence alignment processing [110]. In this latter case, the choice of MonetDB relied on previous performance tests on genome sequences [110], on response time of queries of spatio-temporal datasets [111] and on multi query optimization tests on large-scale information retrieval [106]. MonetDB delivers high performance when operating on complex and extensive queries [112] containing large amount of data entries. In addition, for our typical data we have compared the performance of SQL databases with MonetDB. Our experiments compare three DBMSs with one and the same set of test queries on the same database and on the same hardware platform. Importing the database in the different DBMSs resulted in a different size for each DBMS. These values are shown in Table 2; MonetDB uses compression [113] and therefore it is considerably smaller compared to the other two. Moreover, the results in term of execution time of all the three queries unequivocally shows that the best performance is accomplished with the MonetDB DBMS. Queries (Appendix B) were conducted on an Intel(R) Xeon(R) Processor X5355 @ 2.66 GHz, 32 GB memory, Ubuntu OS v.16.04.4 LTS server. For all experiments, query tests were based on MonetDB version 11.27.13, MySQL version 5.7.21 and SQLite version 3.11.0.

**Table 2:** The results corresponding to the Query Tests conducted on different DBMSs. We have performed five runs for each query and taken the mean and standard deviation (SD) value. The results are displayed in seconds (unit).

Query tests conducted on different DBMS			
	SQLite	MySQL	MonetDB
DBMS size (MB)	89	136	63
Query			
Q1: Mean	0.242	0.238	0.018
Q1: SD	0.009	0.493	0.007
Q2: Mean	0.858	8.606	0.018
Q2: SD	0.005	0.005	0.004
Q3: Mean	0.918	0.116	0.015
Q3: SD	0.003	0.005	0.001

#### 4.2.2 Table filling

The values of the tables of the database schema were transposed to table templates by using the SQL-Python module (v.1.2.5.). Subsequently, the populated tables are put in MonetDB using the MonetDB MAPI library (v.1.4.0). Currently version 11.27.13 of MonetDB is used.

### 4.3 Database management: PROFUNGIS post-processing method

The PROFUNGIS pipeline generates a unique set of ZOTU representatives for every individual run file (SRR) of each sample (SRS). In order to incorporate ZOTUs amongst samples and thus updating MDDB with new samples, an automatic approach has been developed (https://github.com/naturalis/mycodiversity/tree/master/PROFUNGIS_post_processing). A ZOTU of a sample will be included in the database if it has not been detected yet in previous samples, and a new record in MDDB will be created to include the sequence. In sequential fashion, the ʻcontainsʼ relation is updated so that the mapping of the new ZOTU representative to the original sample is incorporated in the database. Contrarily, if there is a match of the ZOTU to an existing record, it will be excluded from the ZOTU representative list and only the ‘contains’ relationship will be updated, such to map the ZOTU to the new sample. PROFUNGIS post processing pipeline execution depends on the size of the ZOTU Reference table. In Table 3 we present an indication of performance times of our PROFUNGIS post processing method. For each update step, we consider the incorporation to the reference dataset with 100 processed files (FASTA format) containing ZOTUs generated by PROFUNGIS. Each FASTA file contains an average range of 400–500 ZOTU sequences. For update 1, the upload included the insertion of 47,529 ZOTUs in an initial empty ZOTU reference set. This generated the set of 35,981 ZOTU representatives which were stored in the distinct RefSequence set. The number of ZOTUs after the update decreased due to detection of identical sequences, that is, common ZOTUs. The contains set takes into account this aspect thus keeping track of which ZOTUs are shared among the samples of origin. The succeeding updates follow, for which another set of 100 FASTA files are introduced in the system every time. The sequential updates are shown in Table 3; the execution time (HH:MM:SS unit) for the method to detect existing ZOTUs in MDDB increases as the RefSequence table grows. All update tests were performed on an 8 × 2.7 GHz Intel Xeon 5150 Processor, 16 GB, running Debian 7 cluster node.

**Table 3:** Post processing update steps for the incorporation of additional FASTA files containing processed ZOTU sequence. Each update includes 100 FASTA files (average 426 ZOTUs/file) obtained from 100 distinct samples of a study. The size displayed are for csv format of the Reference ZOTU and Contain datasets while the execution time is displayed in HH:MM:SS format.

Post processing PROFUNGIS						
Run	Input			Output		Execution Time
	ZOTUs in FASTA Files	ZOTUs in Reference	Records in contains	ZOTUs in Reference	Records in contains	
Update 1	47529	empty	empty	35981	48086	00:01:01
	12,5 MB			9.9 MB	13,6 MB	
Update 2	43481	35981	48086	73699	91567	00:02:05
	11,4 MB	9.9 MB	13,6 MB	20,2 MB	26 MB	
Update 3	42508	73699	91567	110289	134074	00:04:36
	11,2 MB	20,2 MB	26 MB	30,2 MB	38 MB	
Update 4	42374	110289	134074	144193	177005	00:05:20
	11,1 MB	30,2 MB	38 MB	39,5 MB	50,2 MB	
Update 5	37535	144193	177005	172463	213984	00:06:43
	9.9 MB	39,5 MB	50,2 MB	47,3 MB	60,7 MB	

## 5 Results

Currently MDDB contains literature metadata obtained from 25 articles published in 2010–2017 (Table 4). The articles that provide SRA submissions, contain over 37 Gb of raw sequence data from which we have currently processed 2.67 Gb for the incorporation of ZOTUs in the RefSequence table. The processed data generated over 100,000 ZOTU representatives as Fungi, covering 512 samples collected in 38 countries worldwide. PROFUNGIS does rely on the input of four mandatory parameters. Two of these parameters (the SRR accessions and the SRA Experiments (SRX) attribute Platform used in the experiment setup) are provided directly from the data acquisition pipeline, while the primers can be provided in the SRX Design description.

**Table 4:** Type and amount of data contained in MDDB.

Data type	Total
Articles	25
SRA submissions	21
SRA Studies (SRP)	21
454 GS	16
Illumina	3
Ion Torrent	2
SRA Experiments (SRX)	4065
SRA Run Files (SRR)	4470 (37.65 Gb)
SRR processed	511 (2.67 Gb)
Raw sequences processed	3037390
ZOTUs generated	172463
ZOTUs assigned to Fungi	110910
SRA Samples (SRS)	3997
SRA SRS curated	512

Also the marker sequenced is never found in the experimental metadata. Therefore, we have currently processed the SRR files linked to SRX records that do provide the set of primers used within the SRX Design. For obtaining the rest of set of primers necessary to process sequence data obtained from other studies, we rely on searching for this information in the PDFs of the publications. For this approach, we have created a reference primer dataset which contain the primer names, the primer sequences, name of marker and aliases for the primer names. This primer reference table contains the list of universal primers used for ITS amplicons [114] and can be further extended with the primers described in most recent publications. These reference terms will define the set of the relevant regular expressions to search. A PDF-parser such as PDFMiner [115] or PyPDF2 [81], will allow to detect the matches of the defined regular expressions in the publications. This idea will be incorporated in the MDDB platform. Set of queries have been designed to retrieve datasets of interest. In this section we present examples of the type of data we can retrieve regarding fungal biodiversity and distribution.

### 5.1 Fungal biodiversity data

The hierarchical implementation of GeoNames applied to the location fields of the samples made the incorporation of countries to a large scale (i. e., continent) possible. As an illustration (Table 5), the diversity of major fungal groups in each major global region is retrievable.

**Table 5:** Amount of ZOTUs assigned for each Phylum in every continental region.

Phylum	Africa	Asia	Europe	North America	Oceania	South America
Basidiomycota	3940	10573	20462	3865	8404	4117
Ascomycota	3862	9499	15563	3049	9542	6783
Mortierellomycota	207	1244	5331	505	1000	708
Unidentified	135	176	139	68	186	103
Mucoromycota	104	423	2479	338	804	269
Chytridiomycota	32	91	91	13	93	30
Rozellomycota	28	117	173	62	111	51
Glomeromycota	12	13	14	12	31	0
Other	8	41	48	22	28	6

**Figure 4:**
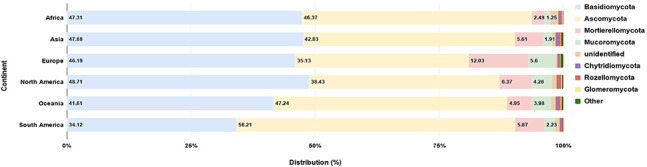
Major phylum ZOTU representatives for each continent.

The taxonomic classification provided in MDDB allows to group all ZOTU representatives in each high taxonomic rank (phylum). As a result, for each continent, Basidiomycota turned out to have the most ZOTU representatives (Figure 4), with the exception of Oceania and South America, were Ascomycota where more abundant in terms of diversity.

### 5.2 Fungal distribution data

**Figure 5:**
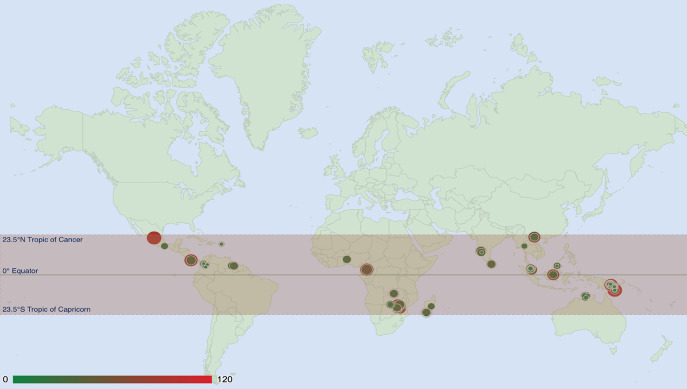
Diversity of ZOTUs belonging to the Russulaceae family and their distribution display in a spatial range. Map display by using Marker GeoCharts (http://developers.google.com/chart/interactive/docs/gallery/geochart).

The taxonomic hierarchy and the curated coordinate values of samples deposited in MDDB allow to display diversity patterns and distribution of specific taxonomic group of fungi. As an example, in Figure 5 we illustrate the distribution of the Russulaceae family within the spatial range among the tropic of Cancer and tropic of Capricorn. The map also displays the diversity of the family based on the distinct ZOTU occurrences for each plot. Queries can be further extended for obtaining set of ZOTU sequences observed in samples and display taxonomic distributions in refined environmental criterias, for example by comparing species richness among biomes.

### 5.3 Shared ZOTUs among continents

The adaptation of a uniform pipeline to process HTS data, has allowed the incorporation of ZOTUs among samples. MDDB permits not only to observe fungal composition within a sample, but also allows to observe which global regions and environmental conditions have the most similar fungal community composition as revealed by the shared fungal sequences observed in the different locations. Table 6 shows the type of data associated with each continent and the number of ZOTUs in the current version of MDDB. The data is currently biased because most of the samples included in MDDB are from Asia, Europe and Oceania. With MDDB, we are able to retrieve the amount of ZOTUs for a given location and compare it with the rest of the globe. Europe and Asia share a comparatively high number of shared ZOTUs between the rest of the globe.

**Table 6:** ZOTUs representatives for each continent and common ZOTUs among continents.

Amount	Globe	Africa	Asia	Europe	North America	Oceania	South America
Number of countries	38	5	11	10	5	3	4
Number of samples	512	35	85	217	28	88	59
Number of ZOTUs	172463	13355	33178	68566	11414	31247	19324
Number of ZOTUs shared betweeen continents		497	2192	2191	926	1306	929

**Figure 6:**
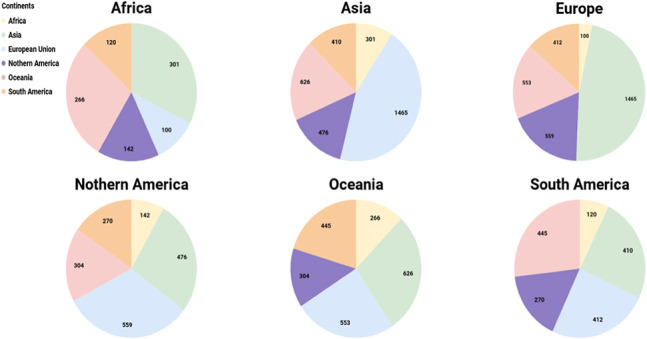
Overlap of ZOTUs among continents.

Meiser et al., [71], presented the amount of shared OTUs among studies and with a similar approach, MDDB provides the possibility to display the fungal similarities and comparisons among locations and compare a specific region composition with the rest of the globe. The pie-charts in Figure 6, illustrate the geographical distribution of the ZOTUs that occur on more than one continent. The number of samples and number of ZOTUs included in the continents Oceania and Asia are more similar to each other compared to the other continents, while the number of shared ZOTUs among Asia and Europe is considerably high compared to the shared ZOTUs between Europe and Oceania. Appendix C shows the queries constructed to generate the output results displayed in this section.

## 6 Discussion

The FAIR principles [86] have been leading in the design, implementation and deployment of the MDDB. An important result of our framework, we provide reliable high quality data in a feasible manner so as to facilitate the community for fungal biodiversity discovery. Additionally, MDDB allows investigating the environmental contributions to fungal distribution and biodiversity. The consistent structure of MDDB assures maintaining the integrity of data pertaining publicly available metabarcoding studies. For our implementation, the FAIR principles for data enrichment with standard terms and methods as well as controlled vocabularies and ontologies are applied. This assures good data integration and data longevity. It is a crucial factor in the further development of MDDB.

### 6.1 Data feasibility and accessibility

Accessing data that is stored across different public sources in a feasible manner is one of the keys for leading to knowledge discoveries. The tools Entrez direct E-utilities, NCBI Run browser and Run selector [116] permit data selection and data retrieval associated with SRA. However, these utilities are only efficient when the Entrez Unique Identifiers are provided in constructing the search (for example, with using SRA study accession numbers), but the selection becomes challenging when text search is applied. Furthermore, the raw sequence data associated to SRA records is only achievable when SRR accessions numbers are used in combination with the SRA Toolkit. The packages SRAdb [117] and SRAmongo [118] facilitate the access and data retrieval from the SRA database. They also tend to aggregate data from the NCBI interlinked databases (e. g., SRA and PubMed), however data from the publication is retrievable if only the databases cross-reference is interoperable. Regarding interoperability of the NCBI resources (Appendix A, PMID and SRA Cross Reference attributes) there are inconsistencies. For example, by using the NCBI Run selector, the PMID of the associated article was not found in the namespace of Tedersoo’s SRA study [36]. With our data acquisition method (Section 3.1), the SRA study is directly retrievable by using the DOI of the publication. To the best of our knowledge, MDDB is the first to provide the complete mapping of NCBI publications to SRA based on a selected list. The complete mapping has been achieved by retrieving the study identifiers from the PDFs of the publications for which this information is not contained in the associated PubMed. This relationship facilitates the selection of studies and experimental assays by using literature text terms (e. g., article title and keywords within an abstract). Additionally, it allows the direct access of publications describing mycodiversity to its sequencing data (e. g., a list of fungal species detected can be retrieved by a publication title).

### 6.2 Data integration and interoperability

The integration of environmental samples for the exploration of global fungal diversity and distribution can be achieved if metadata retrieved from public domains is formalized, curated and standardized. At the same time uniform methods must be applied to the raw HTS data. Data associated with studies is increasingly made available in public data repositories [119], [120] making this a valuable source for our domain. However, while doing our research, we have observed the lack of standard terms and controlled vocabularies used to describe biological samples in SRA. We showed the potential of mapping location annotations to Geonames terms, ENVO terms to the habitat annotation and apply standardization to the coordinate values (Section 3.2.1). This data enrichment offers the opportunity to increase relationships between geographical regions and fungal communities across diverse environmental conditions, thereby increasing the interoperability among studies.

We believe that extending the application of semantic mappings to submitted data will reduce heterogeneity and increase integration of studies. We want to further enrich MDDB with ENVO vegetation and materials terms (i. e., soil) and assign CheBI [121] terms to the nutrient components, such as carbon and nitrogen and to conditions, for instance to the hydrogen values used for the acidity of the soil. Furthermore, we will apply the Units Ontology [122] to control the formats of units in association to measurements, such as to the concentration and amount of compounds found in the environmental material and to the soil depth and amount of sample collected. Moreover, we will include the application of the ENVO ontological classification for extending the integration of studies by using biomes terms.

Finally, we emphasize the application of text mining tools for the analysis of publications, such as the use of EXTRACT to retrieve possible terms within the text. Usually in a publication there is an extensive description of the habitat. Natural language processing methods will allow the retrieval the NCBITaxon terms (i. e., plant species names) constituting the associate host organisms from which a sample has been collected. The essential parameters for processing the sequence data (i. e., primers, barcode type) can be obtained by searching for the relevant regular expressions in the PDF of the publication as proposed here; these valuable annotations will be included in MDDB in a standard format.

MDDB is the first repository which provides reliable sets of ZOTUs generated by a supportive pipeline for denoising HTS data from the widely used HTS platforms and which have been deposited in SRA. The choice of an uniform methodology allows to directly compare the generated ZOTUs and the taxonomic composition among studies. The low number of shared OTUs among the studies selected by Meiser et al., [71] has suggested high global fungal diversity and indicates that globally distributed taxa may be rare. Increasing the number of SRA samples in MDDB, will allow to identify more shared ZOTUs among studies and determine which fungal communities are highly similar among compared studies.

### 6.3 Data reusability

MDDB is a reliable system to which its enriched metadata is always associated to the provenance and third-party annotations by the use of URIs. It is important to emphasize at this stage that data will be made accessible and retrievable by means of a high-end interface and an API. The prototype user interface (https://mycodiversity.liacs.nl) gives insight of the data contained in MDDB, and is leading to serve as a guide for comparative studies as well as constructing both local and global scale assessments for fungal discoveries. Data are reusable and reproducible, as for now by the use of the tools (https://github.com/naturalis/mycodiversity) and by means of extensions which will be included in the future interfaces.

MDDB will incorporate more SRA studies and consequently include more data. The MonetDB DBMS guarantees high performance on queries against big datasets and this can auxiliate users to efficiently select and retrieve data. It is our intention to allow the navigation of public fungal metabarcoding data in a feasible, straightforward manner, such that the user will not spend unnecessary time in the curation and organization of data [123] and enhance time for fungal analysis.

## 7 Final remarks

The rationale of this paper is to illustrate our framework for the curation and integration of fungal metabarcoding data which is compiled in the MDDB database. With this dedicated database, we now can efficiently retrieve data from collections of fungal metabarcoding studies. The database can be further extended with the latest studies using all the tools that we have presented for integration and curation. The integration and curation processes guaranteed uniformity of the data in MDDB, thereby accomplishing an enhancement of availability of public domain fungal metabarcoding data. The database and the pipelines are now ready for large-scale application.

The further extension of the GUI is complementary to the integration effort. The interface is built from predefined queries. We start with relatively simple predefined queries and as the interface evolves more complex queries can be composed. This is part of the future work, some of the ideas are already implemented. The usability within the community is leading the further development of the user interface.

All considered, this database is ready to support and facilitate large-scale research in fungal biodiversity.

## Appendix A

**Table A.1:** Selection of publications describing metabarcoding studies.

Article	Year	PMID	Study	SRA Study Title	PMID C.R.	SRA C.R.
1. Fungal biogeography. Global diversity and geography of soil fungi.	2014	25430773	SRP043706	Global fungal diversity	Y	Null
DOI: 10.1126/science.1256688						
2. New primers to amplify the fungal ITS2 region–evaluation by 454-sequencing of artificial and natural communities.	2012	22738186	SRP012868	Design and test new primers to be used to amplify the fungal ITS2 region by targeting sites in the 5.8S encoding gene	Null	Null
DOI: 10.1111/j.1574-6941.2012.01437.x						
3. Strong altitudinal partitioning in the distributions of ectomycorrhizal fungi along a short (300 m) elevation gradient.	2015	25655082	SRP043982	Root associated fungi Metagenome	Y	Null
DOI: 10.1111/nph.13315						
4. Roots and associated fungi drive long-term carbon sequestration in boreal forest.	2013	23539604	SRP016090	The Island Project – Fungal communities in boreal forest soils	Null	Null
DOI: 10.1126/science.1231923						
5. ITS1 versus ITS2 as DNA metabarcodes for fungi.	2013	23350562	SRP026239	Analysis of ITS1 and ITS2 regions for barcoding fungal specimens	Null	Y
DOI: 10.1111/1755-0998.12065						
6. Dispersal in microbes: fungi in indoor air are dominated by outdoor air and show dispersal limitation at short distances.	2013	23426013	SRP015917	Fungal ITS amplicons from airborne household dust	Null	Null
DOI: 10.1038/ismej.2013.28						
7. Quantifying microbial communities with 454 pyrosequencing: does read abundance count?	2010	21050295	SRP001800	Pyrosequencing of Global House Dust	Null	Y
DOI: 10.1111/j.1365-294X.2010.04898.x						
8. Indoor fungal composition is geographically patterned and morediverse n temperate zones than in the tropics.	2010	20616017	SRP001800	Pyrosequencing of Global House Dust	Null	Y
DOI: 10.1073/pnas.1000454107						
9. Spatio-temporal dynamics of soil bacterial communities as a function of Amazon forest phenology.	2018	29531240	GENBANK	Null	Null	Null
DOI: 10.1038/s41598-018-22380-z						
10. Phylogenetic relatedness explains highly interconnected and nested symbiotic networks of woody plants and arbuscular mycorrhizal fungi in a Chinese subtropical forest.	2017	28207957	SRP042134	Plant roots in subtropical forest Metagenome	Y	Null
DOI: 10.1111/mec.14061						
11. Convergence and contrast in the community structure of Bacteria, Fungi and Archaea along a tropical elevation-climate gradient.	2017	28402397	SRP102378	Hawaii Soils Raw sequence reads	Null	Null
DOI: 10.1093/femsec/fix045						
12. FUNGAL SYMBIONTS. Global assessment of arbuscular mycorrhizal fungus diversity reveals very low endemism.	2015	26315436	ERP010906	Global assessment of arbuscular mycorrhizal fungus diversity reveals very low endemism	Null	Null
DOI: 10.1126/science.aab1161						
13. Arbuscular mycorrhizal interactions of mycoheterotrophic Thismia are more specialized than in autotrophic plants.	2017	27739593	SRP083901	Arbuscular mycorrhizal interactions of mycoheterotrophic Thismia are more specialized than autotrophic plants	Null	Null
DOI: 10.1111/nph.14249						
14. A phosphorus threshold for mycoheterotrophic plants in tropical forests.	2017	28148744	SRP076949	Study of fertilization, litter addition, and soil phosphorus effects on fungal and arbuscular mycorrhizal fungal communities in Panamanian tropical forest	Null	Null
DOI: 10.1098/rspb.2016.2093						
15. Fungal-host diversity among mycoheterotrophic plants increases proportionally to their fungal-host overlap.	2017	28515898	SRP082976	Host diversity increases proportionally to host overlap among mycoheterotrophic plants	Null	Null
DOI: 10.1002/ece3.2974						
16. Consistent responses of soil microbial communities to elevated nutrient inputs in grasslands across the globe.	2015	26283343	SRP052716	Soil marker gene sequences across the Nutrient Network	Y	Null
DOI: 10.1073/pnas.1508,382,112						
17. Severe plant invasions can increase mycorrhizal fungal abundance and diversity.	2013	23486251	SRA037764	Not Accessible	Null	Null
DOI: 10.1038/ismej.2013.41						
18. Host identity is a dominant driver of mycorrhizal fungal community composition during ecosystem development.	2015	25640965	SRP045608	Host preference drives mycorrhizal fungal niche differentiation throughout ecosystem development	Y	Null
DOI: 10.1111/nph.13226						
19. Fungal community analysis by large-scale sequencing of environmental samples.	2005	16151147	GENBANK	Null	Null	Null
DOI: 10.1128/AEM.71.9.5544-5550.2005						
20. Comparison and Validation of Some ITS Primer Pairs Useful for Fungal Metabarcoding Studies	2014	24933453	SRP026207	Pioneer pine forest soil Targeted Locus (Loci)	Null	Null
DOI: 10.1371/journal.pone.0097629						
21. Lack of host specificity leads to independent assortment of dipterocarps and ectomycorrhizal fungi across a soil fertility gradient.	2015	26032408	SRP057798	Root associated fungi Targeted loci environmental	Null	Y
DOI: 10.1111/ele.12459						
22. Meta-analysis of deep-sequenced fungal communities indicates limited taxon sharing between studies and the presence of biogeographic patterns.	2017	24111803	Multi	Null	Null	Null
DOI: 10.1111/nph.12532						
23. Sequence Depth, Not PCR Replication, Improves Ecological Inference from Next Generation DNA Sequencing.	2014	24587293	SRP035367	DOB_RepEx Targeted Locus (Loci)	Null	Y
DOI: 10.1371/journal.pone.0090234						
24. Strong linkage between plant and soil fungal communities along a successional coastal dune system.	2016	27411980	SRP059280	Ectomycorrhizal fungal communities in a relic foredune plain	Null	Null
DOI: 10.1093/femsec/fiw156						
25. Fungal endophyte communities reflect environmental structuring across a Hawaiian landscape.	2012	22837398	SRP013695	Fungal ITS1 amplicon library from environmental samples Targeted Locus (Loci)	Null	Null
DOI: 10.1073/pnas.1209872109						

## Appendix B: Example queries used for comparing the performance in different DBMS, Section 4.2.1, Table 2

### ZOTU representatives in single location

#### Query 1 (Q1): provide all sequence representatives (ZOTUs) from a specific sample

SELECT DISTINCT RS.Sequence_pk, RSM.Amount

FROM SampleMetadata1 SM, RefSampleSequence RS, RefSeqSampleMapping RSM

WHERE SM.Sample_id = ‘SRS651086’ AND SM.Sample_pk = RSM.Sample_pk AND

RSM.Sequence_pk = RS.Sequence_pk;

530 tuples

### Specific ZOTUs compare to another location

#### Query 2 (Q2): provide ZOTUs which are specific in one region and not found in another region of the same country.

SELECT DISTINCT RS.Sequence_pk, RS.RefSeq, SM.Location_name

FROM SampleMetadata1 SM, RefSampleSequence RS, RefSeqSampleMapping RSM

WHERE SM.Country_name = ‘Estonia’ AND SM.Location_name = ‘Jarvselja’

AND SM.Sample_pk = RSM.Sample_pk AND RSM.Sequence_pk = RS.Sequence_pk

AND RS.RefSeq

NOT IN (SELECT RS2.RefSeq

FROM SampleMetadata1 SM2, RefSampleSequence RS2, RefSeqSampleMapping RSM2

WHERE SM2.Country_name = ‘Estonia’ AND SM2.Location_name = ‘Satakunta’

AND SM2.Sample_pk = RSM2.Sample_pk AND RSM2.Sequence_pk = RS2.Sequence_pk;

5075 tuples

### Observance of a specific fungal class

#### Query 3 (Q3): provide a list of locations (i.e. countries) where particular ZOTUs belonging to a specific class (i.e. Fungal family) are detected.

SELECT DISTINCT RS.Sequence_pk, SM.Country_name, URS.GenusName

FROM SampleMetadata1 SM, RefSampleSequence RS, RefSeqSampleMapping RSM,

UniteRefSequence URS, AssignTaxa AT

WHERE URS.GenusName = ‘Lactarius’ AND URS.RefSH_pk = AT.RefSH_pk AND

AT.Sequence_pk = RS.Sequence_pk AND RS.Sequence_pk = RSM.Sequence_pk

AND RSM.Sample_pk = SM.Sample_pk;

339 tuples

## Appendix C: MonetDB queries used for genereating the outputs shown in Section 5 Results

### Fungal diversity query

#### Example 1: Retrieve ZOTUs for each phylum for a continent

SELECT DISTINCT RTDB.Phylum_name as phylum, count(DISTINCT AT.Refsequence_pk) AS zotus

FROM Sample as S, Contain as C, RefSequence as RS, AssignTaxa as AT,

RefTaxonomicDB as RTDB

WHERE S.country_continent = ‘Europe’ AND S.Sample_pk = C.Sample_pk AND

C.Refsequence_pk = RS.Refsequence_pk AND RS.Refsequence_pk = 

AT.Refsequence_pk AND AT.percentage_similarity >70 AND

AT.Refsequence_taxonomic_pk = RTDB.Refsequence_taxonomic_pk

GROUP BY RTDB.Phylum_name

### Fungal distribution query

#### Example 2: Select a specific family and provide the ZOTU representatives of the family within the spatial range between the Tropic of cancer and Tropic of Capricorn

SELECT DISTINCT S.Sample_pk AS plot_id, S.sample_location_country AS

country, S.location_split2 AS location, ABS(S.sample_latitude) AS

dist_from_equator, count(C.Refsequence_pk) as fam_div, count(distinct

RTDB.sh_unite_id) as unite_div

FROM SampleMD as S, Contain as C, RefSequence as RS, AssignTaxa as AT,

RefTaxonomicDB as RTDB

WHERE RTDB.kingdom_name LIKE ‘Fungi%’ AND RTDB.family_name LIKE

‘Russulaceae%’ AND RTDB.Refsequence_taxonomic_pk = 

AT.Refsequence_taxonomic_pk AND AT.Refsequence_pk = RS.Refsequence_pk AND

RS.Refsequence_pk = C.Refsequence_pk AND C.Sample_pk = S.Sample_pk AND ABS(S.sample_latitude) <= 23.5

GROUP BY (S.Sample_pk), S.sample_location_country, S.location_split2,

RTDB.family_name, S.sample_latitude

ORDER BY fam_div DESC

### Shared ZOTUs query

#### Example 3: Retrieving shared data among studies Query1 retrieves ZOTUs grouped for each continent. Query2 displays how to retrieve data shared among attributes, in this case ZOTUs among continents.

SELECT DISTINCT S.country_continent AS continent, count(DISTINCT

S.country_geoname_pref_en) AS countries, count(DISTINCT S.Sample_pk) AS

samples, count(DISTINCT RS.RefSequence_pk) AS zotus

FROM SampleMD S, Contain C, RefSequence RS

WHERE S.Sample_pk = C.Sample_pk AND C.RefSequence_pk = RS.RefSequence_pk

GROUP BY S.country_continent

SELECT DISTINCT S.country_continent AS continent, count(DISTINCT

RS.RefSequence_pk) AS zotus FROM SampleMD S, Contain C, RefSequence RS

WHERE S.Sample_pk = C.Sample_pk AND C.RefSequence_pk = RS.RefSequence_pk

AND RS.RefSequence_pk IN (SELECT RS2.RefSequence_pk AS zotus2

FROM SampleMD S2, Contain C2, RefSequence RS2

WHERE S2.country_continent = ‘South America’ AND S2.Sample_pk = 

C2.Sample_pk AND C2.RefSequence_pk = RS2.RefSequence_pk)

GROUP BY S.country_continent
